# Preoperative imaging of glioblastoma patients using hyperpolarized ^13^C pyruvate: Potential role in clinical decision making

**DOI:** 10.1093/noajnl/vdab092

**Published:** 2021-06-28

**Authors:** Jun Chen, Toral R Patel, Marco C Pinho, Changho Choi, Crystal E Harrison, Jeannie D Baxter, Kelley Derner, Salvador Pena, Jeff Liticker, Jaffar Raza, Ronald G Hall, Galen D Reed, Chunyu Cai, Kimmo J Hatanpaa, James A Bankson, Robert M Bachoo, Craig R Malloy, Bruce E Mickey, Jae Mo Park

**Affiliations:** 1 Advanced Imaging Research Center, The University of Texas Southwestern Medical Center, Dallas, Texas, USA; 2 Department of Neurosurgery, The University of Texas Southwestern Medical Center, Dallas, Texas, USA; 3 Department of Radiology, The University of Texas Southwestern Medical Center, Dallas, Texas, USA; 4 Department of Pharmacy Practice, Texas Tech University Health Sciences Center, Dallas, Texas, USA; 5 GE Healthcare, Dallas, Texas, USA; 6 Department of Pathology, The University of Texas Southwestern Medical Center, Dallas, Texas, USA; 7 Department of Imaging Physics, The University of Texas MD Anderson Cancer Center, Houston, Texas, USA; 8 Department of Neurosurgery and Neurotherapeutics, The University of Texas Southwestern Medical Center, Dallas, Texas, USA; 9 Department of Internal Medicine, The University of Texas Southwestern Medical Center, Dallas, Texas, USA; 10 Department of Electrical and Computer Engineering, The University of Texas at Dallas, Richardson, Texas, USA

**Keywords:** bicarbonate, glioblastoma, hyperpolarized, preoperative, pyruvate

## Abstract

**Background:**

Glioblastoma remains incurable despite treatment with surgery, radiation therapy, and cytotoxic chemotherapy, prompting the search for a metabolic pathway unique to glioblastoma cells.^13^C MR spectroscopic imaging with hyperpolarized pyruvate can demonstrate alterations in pyruvate metabolism in these tumors.

**Methods:**

Three patients with diagnostic MRI suggestive of a glioblastoma were scanned at 3 T 1–2 days prior to tumor resection using a ^13^C/^1^H dual-frequency RF coil and a ^13^C/^1^H-integrated MR protocol, which consists of a series of ^1^H MR sequences (T_2_ FLAIR, arterial spin labeling and contrast-enhanced [CE] T_1_) and ^13^C spectroscopic imaging with hyperpolarized [1-^13^C]pyruvate. Dynamic spiral chemical shift imaging was used for ^13^C data acquisition. Surgical navigation was used to correlate the locations of tissue samples submitted for histology with the changes seen on the diagnostic MR scans and the ^13^C spectroscopic images.

**Results:**

Each tumor was histologically confirmed to be a WHO grade IV glioblastoma with isocitrate dehydrogenase wild type. Total hyperpolarized ^13^C signals detected near the tumor mass reflected altered tissue perfusion near the tumor. For each tumor, a hyperintense [1-^13^C]lactate signal was detected both within CE and T_2_-FLAIR regions on the ^1^H diagnostic images (*P* = .008). [^13^C]bicarbonate signal was maintained or decreased in the lesion but the observation was not significant (*P* = .3).

**Conclusions:**

Prior to surgical resection, ^13^C MR spectroscopic imaging with hyperpolarized pyruvate reveals increased lactate production in regions of histologically confirmed glioblastoma.

**Key Points**

- Preoperative imaging of glioblastoma patients using HP pyruvate was performed.
- HP pyruvate adds a metabolic axis to the radiographic diagnosis of glioblastoma.
- HP ^13^C MRI is feasible in the routine clinical workflow for glioblastoma patients.

**Importance of the Study**

Warburg’s hypothesis postulates that cancer cells rely largely on aerobic glycolysis for energy production. However, previous ^13^C NMR spectroscopy studies reported that metabolic alteration in glioblastomas is more complex as they also use oxidative metabolic pathways to provide the energy required by the glioblastoma cell for growth and migration although aerobic glycolysis is present in these tumors. This study demonstrates the feasibility of using ^13^C MR imaging with hyperpolarized pyruvate in the preoperative evaluation of glioblastoma and validates the ^13^C imaging results with surgically removed tumor samples. ^13^C MR spectroscopic imaging with hyperpolarized pyruvate has the potential to provide real-time metabolic data to be used in the preoperative evaluation of a suspected glioblastoma, in planning surgical procedures, and in monitoring the metabolic response to adjuvant therapy.

The best available treatment for a glioblastoma, a maximum safe resection followed by adjuvant radiation and temozolomide, typically provides a temporary cessation of tumor growth followed by treatment resistant progression or recurrence.^1^ A better understanding of the metabolic processes underlying the initiation, growth, and recurrence of glioblastoma would benefit both clinical decision making and the search for more effective treatment strategies.

Initial studies of glioblastoma metabolism utilized the administration of labeled substrates to cells growing in tissue culture or to heterotopic tumors, followed by isotopomer analysis using mass spectroscopy or nuclear magnetic resonance (NMR). More recently, the infusion of glucose labeled with the stable isotope ^13^C at the time of glioblastoma surgery, followed by NMR analysis of the resected tumor, has confirmed the presence of aerobic glycolysis in these tumors^2^ but has revealed that they also metabolize glucose^3^ and other substrates, such as acetate,^4^ in the tricarboxylic acid (TCA) cycle. These studies obviously require surgical intervention to provide metabolic information.

Attempts to noninvasively study glioblastoma metabolism in vivo have evolved from proton magnetic resonance (MR) spectroscopy of *N*-acetyl aspartate and choline, molecules found in sufficient concentration in tumors and in normal brain to be reliably detected at 1.5 Tesla (T). The optimization of proton MR spectroscopy now permits the noninvasive detection of important glioblastoma metabolites such as lactate, glutamate, and glycine, but these measurements are hindered by signal-to-noise issues even at higher field strengths due to the relatively low concentrations of these molecules and require complex editing techniques for quantification. Moreover, the method focuses on the static state or the pool size of the metabolite rather than the enzyme activity.

In an effort to improve the signal produced by low abundance metabolic substrates and their downstream products, the stable isotope ^13^C can be “hyperpolarized” using dynamic nuclear polarization, thereby dramatically enhancing the MR detectable signals of substrates and metabolites that have been labeled with this isotope.^5^ In this fashion, MR spectroscopy of hyperpolarized [1-^13^C]pyruvate can determine in vivo what fraction of pyruvate is metabolized to bicarbonate via pyruvate dehydrogenase (PDH), and what fraction is converted to lactate via lactate dehydrogenase (LDH).^6^ Administered [1-^13^C]pyruvate is enzymatically reduced to [1-^13^C]lactate in glycolysis or converted to acetyl-CoA and carbon dioxide, detected in vivo as [^13^C]bicarbonate under physiological pH, as it enters the mitochondria, Figure 1. Therefore, [1-^13^C]lactate observed following the injected hyperpolarized [1-^13^C]pyruvate has been suggested as a biomarker of glycolysis with measurements correlated with LDH activity. In contrast, [^13^C]bicarbonate detection in an hyperpolarized [1-^13^C]pyruvate study is a surrogate marker for oxidative phosphorylation as the majority of acetyl-CoA undergoes oxidative phosphorylation in the brain. Preclinical studies using hyperpolarized [1-^13^C]pyruvate connected the altered LDH and PDH activities of glioblastoma to altered [1-^13^C]lactate and decreased [^13^C]bicarbonate, respectively.^7,8^ Both preclinical^9–15^ and clinical^16,17^ studies have suggested that MR spectroscopy with hyperpolarized [1-^13^C]pyruvate might be used to monitor therapeutic responses in gliomas.

**Figure 1.**
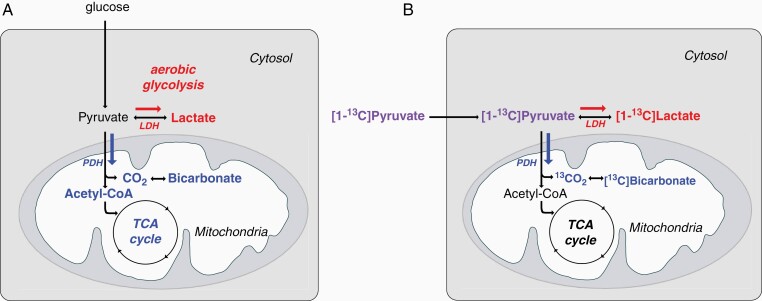
Glioblastoma metabolism. (A) Pyruvate can be metabolized via lactate dehydrogenase (LDH) to lactate in the presence of oxygen, aerobic glycolysis (red). Pyruvate can also enter the mitochondria where it is metabolized to CO_2_ and acetyl-CoA via pyruvate dehydrogenase (PDH) (blue). (B) Labeled carbon (^13^C) of injected [1-^13^C]pyruvate (purple) becomes [1-^13^C]lactate (red) or [^13^C]bicarbonate (blue).

To date, practical considerations such as the specialized equipment required to produce substrates labeled with hyperpolarized ^13^C, and the very short half-life of the substrates once labeled, have largely limited clinical studies to patients already under treatment. In an effort to characterize pyruvate metabolism in treatment-naïve patients, we studied three patients with suspected, newly diagnosed glioblastoma with hyperpolarized [1-^13^C]pyruvate and correlated those results with data obtained from conventional proton MR imaging, and with the histopathology of the surgically resected tissue samples. We have shown that MR spectroscopy with hyperpolarized [1-^13^C]pyruvate can be incorporated into the preoperative evaluation of a patient suspected to have a glioblastoma without interrupting the clinical workflow and that the images obtained have the potential to inform clinical decision making.

## Materials and Methods

### Patient Recruitment

Three patients (age 63–71 years, two males and one female) with abnormal MR enhancing regions, suggestive of a high-grade glioma, were recruited at the UT Southwestern Medical Center. The patient demographics are summarized in Table 1. The patients underwent MR imaging including spectroscopy with hyperpolarized [1-^13^C]pyruvate 1 or 2 days prior to surgery for the removal of the tumor. The study was approved by the local Institutional Review Board (STU 012017-070, ClinicalTrials.gov ID: NCT03759704). Informed written consent was obtained from all participants. The study was HIPAA compliant and was conducted under an Investigational New Drug approval from the Food and Drug Administration (IND#: 133229).

**Table 1.** Patient Demographics, Diagnosis, and Acquired MR Data

Participant ID	1	2	3
Age	71	63	66
BMI	26.4	25.4	18.7
Sex	M	M	F
Diagnosis	Glioblastoma, IDH^wt^	Glioblastoma, IDH^wt^	Glioblastoma, IDH^wt^
^1^H MRI protocol	ASL, CE T_1_w, T_2_w, MRS	T_2_w	ASL, CE T_1_w, T_2_w

ASL, arterial spin labeling; CE, contrast-enhanced; IDH^wt^, isocitrate dehydrogenase wild type; MRS, MR spectroscopy.

### Hyperpolarization

A SPINlab™ polarizer (GE Healthcare) that operates at ~0.8 K in a 5-T magnet was used for hyperpolarization. Two samples of pyruvic acid were assembled into clinical fluid paths (GE Healthcare) under a sterile environment and simultaneously polarized for each participant. Each pyruvate sample was prepared by mixing 1.47 g of GMP-grade 14-M [1-^13^C]pyruvic acid (Sigma Aldrich) with 27.7 mg of AH111501 radical (Syncom). The pyruvate sample was polarized for 3–4 hours before dissolving with 38 mL of sterile water at 130°C. The hyperpolarized pyruvate solution was immediately mixed with 36.5 mL of buffer media that contains 333-mM TRIS and 600-mM NaOH, resulting in 250-mM of pyruvate concentration, then examined by a dedicated quality control device (GE Healthcare) prior to the injection. In addition, terminal filtering, bubble point test, pH strip confirmation, and volume check were performed after the automated quality control. Hyperpolarized pyruvate (0.1 mmol/kg body weight) was intravenously administered with an injection rate of 5 mL/s, followed by a 25-mL saline flush.

### Study Visit and MR Protocol

**Figure 2.**
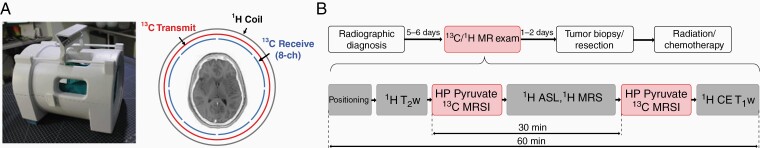
Overall study design using a ^13^C/^1^H-integrated MR protocol. (A) A dual-frequency RF head coil that consists of three concentric coils: ^1^H transmit and receive,^13^C transmit, and eight-channel ^13^C receive. (B) Patients, radiographically diagnosed with suspected GBM, were recruited 1–2 days prior to tumor resection for an ^13^C/^1^H MR exam that includes two injections of hyperpolarized (HP) [1-^13^C]pyruvate and MR spectroscopic imaging (MRSI).

Study participants arrived at the Advanced Imaging Research Center at 9:00 am, following an overnight fast. After confirming informed consent, an electrocardiogram and vital signs were obtained. One intravenous line was placed in the right median cubital vein. The participants were given 48 g of glucose as an oral paste and moved to a clinical 3T 750w Discovery MR scanner (GE Healthcare). The participants were positioned on the scanner table supine with their head in a ^13^C/^1^H dual-frequency RF head coil (Clinical MR Solutions, LLC) that consists of ^1^H transmit/receive, ^13^C transmit, and eight-channel ^13^C receive array in a concentric nested design (Figure 2A).^18^ The ^13^C/^1^H head coil was critical for colocalizing ^1^H and ^13^C imaging slices and for completing the ^13^C/^1^H-integrated MR protocol within 1 hour without swapping ^13^C and ^1^H RF coils.

Study participants were imaged using a ^13^C/^1^H-integrated MR protocol that includes the standard ^1^H MRI scans and ^13^C spiral chemical shift imaging (CSI) with two injections of hyperpolarized [1-^13^C]pyruvate. The two injections were separated by 30 minutes. Overall study timeline and the integrated MR protocol is summarized in Figure 2B. First, each subject was scanned by a three-plane ^1^H localizer, followed by axial T_2_-weighted (T_2_w) fluid-attenuated and inversion recovery imaging (FLAIR; field of view [FOV] = 24 cm × 24 cm, echo time [TE] = 144.7 ms, repetition time [TR] = 8 seconds, inversion time [TI] = 2163 ms, slice thickness = 5 mm, #slices = 16, and flip angle [FA] = 160°). Prior to ^13^C imaging, *B*_0_ inhomogeneity of the target ^13^C slice was minimized using a single-voxel ^1^H point-resolved spectroscopy (PRESS) sequence by adjusting shim gradients. For ^13^C metabolic imaging, a dynamic ^13^C spiral CSI (FOV = 24 cm × 24 cm, in-plane resolution = 1.5 cm × 1.5 cm, slice thickness = 2.5–3 cm, variable FA up to 30° per timepoint, #timepoints = 16, TR = 5 seconds, seven spatial interleaves of spiral readout, spectral width = 814 Hz, and 48 echoes) was used with a bolus injection of hyperpolarized [1-^13^C]pyruvate.^19^ The ^13^C scan was initiated 5 seconds after the start of injection. After the first ^13^C acquisition, T_1_-weighted (T_1_w) FLAIR (FOV = 24 cm × 24 cm, TE = 9.4 ms, TR = 3109 ms, TI = 1215 ms, slice thickness = 5 mm, #slices = 16, and FA = 142°), a 3D pseudo-continuous arterial spin labeling (ASL) with stack of spiral readouts (FOV = 24 cm × 24 cm × 16.2 cm, TE = 9.9 ms, TR = 6733 ms, post-labeling delay = 2025 ms, #averages = 3, matrix size = 512/interleave, #interleaves = 8, and FA = 90°), and ^1^H PRESS MRS (TE = 101 ms, TR = 2 seconds, #averages = 128, voxel size = 2 cm × 2 cm × 2 cm, spectral width = 2500 Hz, and #spectral points = 2048) were acquired during the 30-minute interval between injections. The ASL was performed to identify vascular imbalance of the tumor regions. Finally, the dynamic ^13^C spiral CSI was repeated with another injection of hyperpolarized [1-^13^C]pyruvate. To avoid any T_1_-shortening effect of gadolinium on hyperpolarized ^13^C signals, contrast-enhanced (CE) T_1_w spin-echo (SE; FOV = 24 cm × 24 cm, TE = 12 ms, TR = 700 ms, and FA = 90°) was acquired after both pyruvate injections.

After completion of the study, participants returned to the procedure room for vital signs, a repeat electrocardiogram, removal of the i.v. and observation for 60 minutes. Total duration of the study visit was approximately 3 hours from time of arrival to discharge.

### Data Reconstruction and Analysis

**Figure 3.**
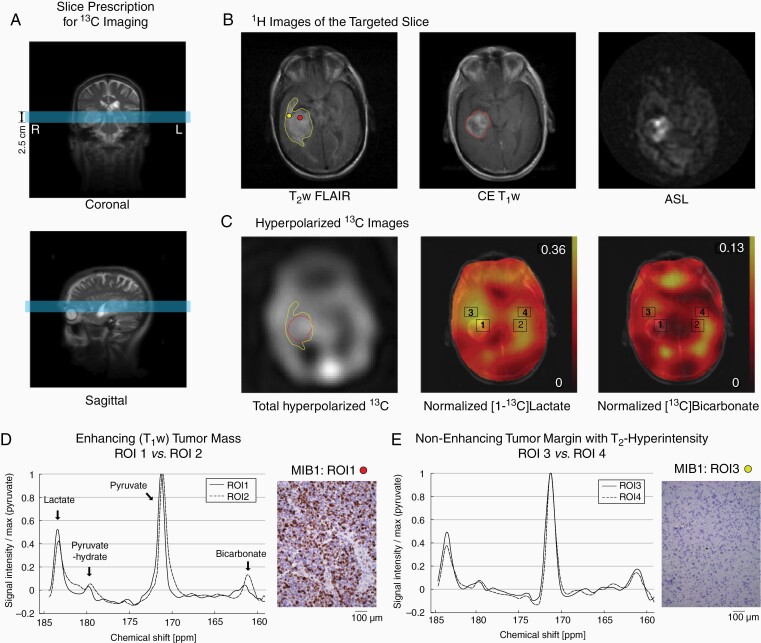
Patient #1. (A) Slice prescription for ^13^C imaging (blue slice). (B) Hyperintense regions in T_2_w FLAIR (yellow contour), CE T_1_w MRI (red contour), and ASL. The red and yellow dots indicate tissue biopsy for MIB1 in D and E. (C) Total hyperpolarized ^13^C signal, and normalized lactate and bicarbonate. (D) ^13^C spectra of enhancing tumor mass (ROI1) and the contralateral NAB (ROI2). (E) ^13^C spectra of nonenhancing but T_2_ hyperintense tumor margin (ROI3) and the contralateral NAB (ROI4).

**Figure 4.**
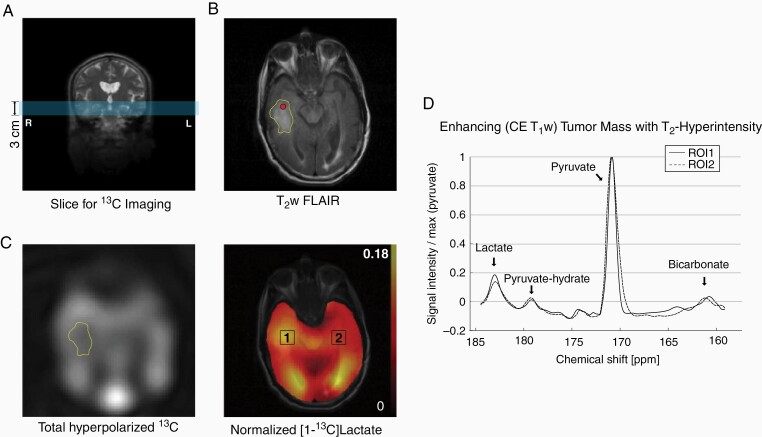
Patient 2. (A) A 3-cm axial image slice (marked in blue) was prescribed to include the tumor mass in the right temporal lobe. (B) Hyperintense region (yellow contour) was detected by T_2_w FLAIR. The red dot indicates tissue biopsy. (C) Higher total hyperpolarized ^13^C signal was detected from the region. (D) Lactate production in the tumor ROI was higher than the contralateral NAB.

**Figure 5.**
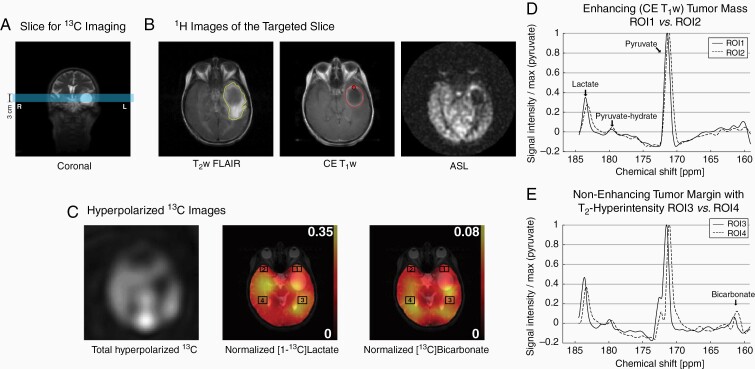
Patient 3. (A) Prescribed slice for ^13^C imaging. (B) Hyperintense tumor margins in T_2_w (yellow contour) and enhancing tumor in CE T_1_w MRI (red contour). Hypoperfusion in the cyst and the posterior T_2_ hyperintense region. The red dot indicates tissue biopsy. (C) Total hyperpolarized ^13^C signal, and normalized lactate and bicarbonate. ^13^C spectra of (D) enhancing tumor (ROI1), (E) nonenhancing tumor margin in the posterior side of the cyst (ROI3) and the contralateral NABs (ROI2, 4).

Depending on the slice thickness of ^13^C imaging slice, 5–6 consecutive corresponding slices of ^1^H images such as T_2_w FLAIR, CE T_1_w SE, and ASL were averaged for drawing regions of interest (ROIs) and for display (Figure 3–5). ^13^C data were reconstructed using MATLAB (Mathworks). The raw data from two hyperpolarized pyruvate injections were averaged, apodized by a 20-Hz Gaussian filter, and zero-filled along the time and spatial domains by a factor of 4, followed by a fast Fourier transform (FFT) along the time domain. Then, the k-space data were gridded onto Cartesian coordinate and 2D inverse FFT was performed in the spatial domain. Metabolite maps of [1-^13^C]pyruvate, [1-^13^C]pyruvate-hydrate, [1-^13^C]lactate, and [^13^C]bicarbonate were generated by integrating the corresponding metabolite peaks in the absorption mode spectra and normalized to the total hyperpolarized ^13^C map, which is sum of pyruvate, pyruvate-hydrate, lactate, and bicarbonate maps, to compensate the spatial heterogeneity in hyperpolarized pyruvate delivery and the RF coil receive profile. For quantitative assessment of each brain metabolite, spectra were averaged over selected ROIs before integrating individual peaks. For display of reconstructed spectra, zeroth- and first-order phase was corrected. For 1H MRS, spectral fitting was performed, with LCModel software,^20^ using an in-house calculated basis set including 2HG, similarly as in a prior study.^21^ Metabolite concentrations were quantified with reference to water. Paired *t* tests were performed to evaluate statistical significance of the difference in the normalized [1-^13^C]lactate and [^13^C]bicarbonate levels between the tumor ROIs and the contralateral normal-appearing brain (NAB) ROIs.

## Results

Each of the participants tolerated the two injections of hyperpolarized pyruvate and the ^13^C/^1^H MR exam without having any adverse effects. Pyruvate concentration (240.8 ± 6.6 mM, *n* = 6), pH (7.9 ± 0.1), polarization level (35.8 ± 3.6%), and radical residue (1.6 ± 0.5 μM) of the hyperpolarized solution were measured. The transfer time from dissolution to injection was 61.6 ± 1.8 seconds.

The first participant was a 71-year-old right-handed man who presented with confusion and dysarthric speech. An MR scan revealed a 4.5-cm heterogeneously enhancing, centrally necrotic mass in the right mesial temporal lobe. A tumor slice was selected for hyperpolarized ^13^C imaging containing the contrast-enhancing mass centrally and peripheral nonenhancing regions of edema/brain infiltration with hyperintensity on T_2_w FLAIR images (Figure 3A). The core tumor mass demonstrated markedly increased relative cerebral blood flow, indicated by ASL perfusion (Figure 3B), with exception of the areas of necrosis, and elevated signal in the total hyperpolarized ^13^C map (Figure 3C). Two ROIs were selected to include the central enhancing component (ROI1) and surrounding nonenhancing T_2_ FLAIR signal abnormality (ROI3), labeled as tumor margin. ROI1 showed increased lactate (relative lactate level to total ^13^C signal = 32.7%) and decreased bicarbonate (relative bicarbonate level = 6.2%) as compared to the contralateral NAB in the left hemisphere (ROI2; relative lactate = 26.4%, relative bicarbonate = 12.4%) (Figure 3D). ROI3 showed increased lactate (relative level = 31.0%) compared to the contralateral NAB region (ROI4, 24.6%), but bicarbonate production was at similar levels (11.5% in ROI3, 12.1% in ROI4) (Figure 3E). 2HG was undetectable with MRS in the tumor (data not shown). The surgical resection of the tumor was performed 2 days after the hyperpolarization exam and was guided by a surgical navigation system. The tumor was reddish gray in color, soft in consistency, and contained thrombosed veins. A complete resection of the enhancing portion of the tumor was achieved. Histologically the tumor was a WHO grade IV glioblastoma with wild-type IDH (IDH^wt^) by immunohistochemistry, with a high mitotic index (MIB1 > 50%, Figure 3D), prominent microvascular proliferation, and geographic necrosis. A specimen obtained from the region of abnormal FLAIR signal adjacent to the tumor revealed scattered atypical cells with reactive astrocytosis as confirmed by GFAP immunostain, but no microvascular proliferation (MIB1 < 1%, Figure 3E) or necrosis. Postoperatively, the patient was treated with a modified chemoradiation protocol with concomitant daily temozolomide. After completing two cycles of adjuvant temozolomide, the patient declined further treatment. At 9-month post-resection, his MR scan revealed no evidence of recurrent tumor, and he was neurologically well.

The second participant was a 63-year-old right-handed man with a history of a WHO grade IV glioblastoma (IDH^wt^) in the left superior parietal lobule, who was identified to have a new tumor mass in the right temporal lobe. The original tumor mass had been completely removed with a postoperative standard chemoradiation protocol 1.5 years prior to the new tumor identification. The right temporal lobe mass progressed after two cycles of temozolomide, but there was no evidence of recurrence of the original tumor in the left parietal lobe, prompting the recommendation that he undergo another surgical resection. As part of his preoperative planning, he underwent a research MR scan with hyperpolarized pyruvate. A 3-cm axial slice that includes the enhancing region of the T_2_w FLAIR image was prescribed for hyperpolarized pyruvate imaging (Figure 4A and B). Elevated hyperpolarized signal was identified in the enhancing region (13.7% larger than the contralateral NAB, Figure 4C). CE T_1_w imaging and perfusion imaging were not acquired during the hyperpolarized session. Therefore, ROI analysis was done in the right temporal tumor mass, based on the T_2_ hyperintensity. Higher hyperpolarized lactate was produced in the tumor mass (relative lactate level = 16.0%) than the NAB (14.8%) (Figure 4C and D). Detection of bicarbonate signal was not reliable due to the low signal-to-noise ratio (SNR). CE T_1_w and perfusion images, acquired from a separate clinical MRI, showed a smaller tumor mass with contrast enhancement and elevated perfusion than the T_2_ hyperintense region (see Supplementary Figure S1). ROI1 was chosen to include the enhancing tumor mass in the CE T_1_w image. A complete removal of the contrast-enhancing portion of the tumor was achieved 2 days after the hyperpolarized study. Histological examination of the enhancing portion of the tumor revealed a highly cellular infiltrating and recurrent glioblastoma (WHO grade IV), with a high mitotic index (MIB1: ~20%), IDH^wt^ and areas of geographic necrosis and microvascular proliferation. Specimens submitted from the brain adjacent to the enhancing portion of the tumor revealed reactive gliosis but were negative for tumor. The right temporal mass recurred and rapidly progressed. The patient passed away 6 months after the surgery.

The third participant was a 66-year-old right-handed woman, who presented with aphasia and was found to have a 3-cm cystic mass with peripheral contrast enhancement in the anterior aspect of her left temporal lobe, with extensive areas of peripheral vasogenic edema. She underwent the hyperpolarized pyruvate research MR scan the day prior to surgery. A 3-cm axial imaging slice was prescribed to include the cystic/necrotic peripherally enhancing mass and peripheral T_2_w FLAIR hyperintense region (Figure 5A and B). ASL demonstrated marked hypoperfusion in the central cystic/necrotic area, a rim of hyperperfusion corresponding to the enhancing component, and milder hypoperfusion peripherally and posteriorly along the areas of edema. Increased conversion of hyperpolarized pyruvate to lactate was observed in the anterior tumor margin (ROI1, relative lactate level = 27.6%) and the T_2_-hyperintensity region posterior to the tumor (ROI3, 31.3%) as compared to the contralateral NABs (ROI2, 23.2%; ROI4, 26.8%) (Figure 5C–E). At surgery, the left middle temporal gyrus was widened but not discolored, and no arterialized veins were noted on the cortical surface. The core centrally necrotic and peripherally enhancing mass was completely removed. On histological examination, specimens obtained from the main tumor mass and the enhancing margin revealed a WHO grade IV glioblastoma, IDH^wt^ by immunohistochemistry. Prominent microvascular proliferation and pseudopalisading necrosis were noted throughout this specimen. Specimens obtained from the left temporal lobe anterior and lateral to the main tumor mass were negative for tumor. A postoperative MR scan documented a complete removal of the contrast-enhancing portion of the tumor. The patient was treated with standard chemoradiation therapy, but her tumor ultimately progressed and she passed away 9.5 months after the surgery.

The relative lactate and bicarbonate levels in the tumor and the contralateral NAB ROIs are summarized in Table 2. Overall, relative lactate levels were higher in the tumor ROIs than the NAB ROIs (*P* = .008) and the difference in relative bicarbonate levels between the tumor and the NAB was not significant (*P* = .3).

**Table 2.** Lactate and Bicarbonate Levels Relative to the Total Carbon Signals

	Region of Interest (ROI)	Relative Lactate		Relative Bicarbonate	
		Tumor	NAB	Tumor	NAB
Patient 1	ROI1, 2	0.327	0.264	0.062	0.124
	ROI3, 4	0.310	0.246	0.115	0.121
Patient 2	ROI1, 2	0.160	0.148	0.071	0.092
Patient 3	ROI1, 2	0.276	0.232	0.111	0.108
	ROI3, 4	0.313	0.268	0.086	0.077

NAB, normal-appearing brain.

Relative lactate and bicarbonate productions measured from individual injections were comparable between the injections (Supplementary Table S1). As compared with measurements from the first injection, relative lactate levels were 6.1 ± 5.4% (*P* = .09) and 0.4 ± 7.2% (*P* = .9) higher in the second measurements in the tumor ROI and the NAB ROI, respectively. Bicarbonate measurements from the second injection were 5.8 ± 13.0% (*P* = .4) and 2.7 ± 10.9% (*P* = .7) higher than the first measurement in the tumor and the NAB, respectively. The contrasts between the tumor and the contralateral NAB ROIs from the two injections were consistent for both lactate and bicarbonate in all subjects.

## Discussion

A more complete understanding of the complex metabolic changes associated with the initiation and progression of a glioblastoma could accelerate the search for more effective treatment strategies. The infusion of ^13^C-labeled glucose or acetate immediately prior to the removal of a glioblastoma, followed by NMR analysis of snap frozen tumor specimens, provides an accurate measure of the metabolism of these substrates by the tumor at one point in time.^2,4^ Studies performed in this fashion have confirmed that glioblastoma cells metabolize glucose to pyruvate and then to lactate even in the presence of oxygen, the so-called Warburg effect, and have shown that glucose is also metabolized via PDH and the TCA cycle.^3^ The present study demonstrates that similar information may be noninvasively obtained prior to surgery using hyperpolarized [1-^13^C]pyruvate and MR spectroscopy.

Using hyperpolarized [1-^13^C]pyruvate, we performed preoperative metabolic imaging of newly diagnosed brain tumors suspected to be high-grade gliomas. At surgery, each of these tumors was histologically confirmed to be a WHO grade IV glioblastoma with IDH^wt^. The total hyperpolarized ^13^C signal, the sum of the signals detected from pyruvate, lactate, and bicarbonate, obtained from the vicinity of the tumor was directly affected by tissue perfusion. Therefore, to compensate for variations in tissue perfusion, lactate and bicarbonate maps were normalized using the total hyperpolarized ^13^C signal. In all three patients, relative lactate levels were increased in both in T_1_ contrast-enhancing regions and in regions with hyperintense T_2_ FLAIR signal. Bicarbonate production was most reliably measured in patient #1, whose relative bicarbonate production was decreased in the enhancing region but not in the nonenhancing regions with T_2_ FLAIR hyperintensity, suggesting the tumor cells at the infiltrating edge rely on both glycolysis and oxidative phosphorylation. This finding may imply that at least some cells in the T_2_ FLAIR hyperintense regions have transitioned to the metabolic state characteristic of malignancy, excess conversion of pyruvate to lactate, providing a justification for greater attention to these regions when planning surgery or radiation therapy. Indeed, it is reportedly that nonenhancing tumor is composed most of nontumor cells with a small component of tumor cells.^22^ The preoperative study of a larger group of patients will be necessary to validate this observation. If validated, the observation of a malignant metabolic phenotype within the region of T_2_ FLAIR hyperintensity surrounding the T_1_ contrast-enhancing portion of the tumor would support an attempt at a supramarginal resection in selected cases.^23,24^

The present study demonstrates that spectroscopy using hyperpolarized [1-^13^C]pyruvate can be readily included in the routine clinical evaluation of a brain tumor patient. However, considering that this imaging method is in early stage of clinical translation and requires additional equipment and hardware such as the hyperpolarizer and the ^13^C RF coil, inclusion of this additional metabolic imaging into the clinical flow needs substantial efforts and investments. In particular, establishing a fast and systematic clinical pipeline between neurosurgeons, neuroradiologists, and the imaging research team is essential for preoperative imaging of glioblastoma patients due to the short lead time between initial diagnosis of glioblastoma and surgery.

For tumors located in brain regions where surgical access entails a high risk of neurologic disability, such as the thalamus, basal ganglia, or sensorimotor area,^25,26^ metabolic information provided by MR imaging with hyperpolarized pyruvate and other substrates may provide helpful diagnostic information. When extended to other key substrates such as acetate and glutamine, this noninvasive and nonradioactive technique could uncover additional metabolic pathways that might be of benefit in the diagnostic evaluation of presumed gliomas^2,4^ and in the search for treatment strategies that might take advantage of metabolic pathways differentially utilized by the tumor.

In addition to extending our observations to a larger cohort of patients, we can suggest several ways in which our observations might be strengthened:

First, detection of bicarbonate was challenging with the current setup. Unlike hyperpolarized pyruvate and lactate signals that were readily detectable, the reliability of bicarbonate measurements was diminished by a low SNR. Other investigators have reported a similar problem, primarily due to the low signal intensity of this substrate.^27–29^ Since high reproducibility of hyperpolarized pyruvate imaging was demonstrated previously in brain and heart studies,^30,31^ we performed two injections of hyperpolarized pyruvate and averaged the recorded signal to improve the SNR of hyperpolarized signals, particularly bicarbonate. We confirmed that the measured lactate and bicarbonate peaks were consistent between the injections in each ROI. There are multiple points in the imaging chain that might enable improved bicarbonate imaging, including the MR pulse sequence, RF coil, and MR scanner performance. Moreover, accelerated quality assurance procedures and dissolution-to-injection time will increase the overall SNR of ^13^C signals. For instance, shortening the delay to 30 seconds will increase the polarized signals by ~50%.

Second, the differences in lactate and bicarbonate between the tumor and the contralateral normal brain region were relatively small in some tumors despite the aggressiveness. This is likely due to the compromised spatial resolution of ^13^C images, resulting in large partial volume effects from the surrounding normal tissue. In this study, we used a spectroscopic imaging method to have full access to ^13^C spectrum at the expense of suboptimal spatial resolution. The large slice thickness was used to achieve reliable SNR of bicarbonate. Spatial resolution can be improved significantly by exploiting spatial-spectral RF excitations in combination with imaging acquisition. This imaging approach will provide more detailed assessment of the tumor metabolism but will require cautious interpretation as spectral-spatial RF pulses are more susceptible to *B*_0_ inhomogeneity and signal contamination from unintended peaks. Sensitivity and specificity of hyperpolarized [1-^13^C]pyruvate for detecting glioblastoma metabolism need to be further evaluated in larger studies with improved spatial resolution.

Third, quantification of hyperpolarized signals was limited in this study and the metabolic signals in the tumor region were only described in relation to the contralateral region of the brain. This is primarily because the distribution of hyperpolarized ^13^C pyruvate and products in healthy brain is not fully investigated. Regional quantification of hyperpolarized signals would be particularly important for midline tumors that do not have a contralateral region for measurement. For direct and quantitative assessment of tumor metabolism in the brain, a metabolic brain atlas needs to be established.

Finally, oral glucose loading is not required. Our decision to use glucose was based on the desire to achieve roughly the same metabolic state across subjects. In some tissues such as the heart and liver, flux of hyperpolarized pyruvate through PDH is quite sensitive to nutrition as well as circulating glucose and ketone levels. The first human studies of the heart using hyperpolarized pyruvate used an oral glucose load prior to imaging.^32^ In these tissues, abundant glucose is associated with higher PDH flux. However, the brain prefers carbohydrates under all conditions except during an extreme fast, and the use of oral glucose has probably minimal effects on the imaging results with hyperpolarized pyruvate.

## Conclusion

This pilot study suggests that preoperative metabolic imaging with hyperpolarized [1-^13^C]pyruvate may be integrated into the clinical evaluation of patients suspected of harboring a glioblastoma. Histologic examination of tissue obtained from brain regions that demonstrated increased lactate and decreased bicarbonate production confirmed the presence of a glioblastoma in those regions. ^13^C MR spectroscopic imaging with hyperpolarized pyruvate has the potential to provide real-time metabolic data to be used in the preoperative evaluation of a suspected glioblastoma, in planning surgical procedures, and in the future, monitoring the metabolic response to adjuvant therapy.

## Supplementary Material

vdab092_suppl_Supplementary_Materials

## Notes

**Authorship Statement.** T.R.P., C.R.M., B.E.M., and J.M.P. designed the study. T.R.P. and B.E.M. recruited patients. J.L., J.R., R.G.H., G.D.R., and J.A.B. prepared clinical fluid paths. J.C., C.E.H., J.D.B., K.D., S.P., C.R.M., and J.M.P. performed imaging studies. J.C., M.C.P., Cha.C., R.M.B., C.R.M., B.E.M., and J.M.P. analyzed the MR data. Chu.C., K.J.H., and R.M.B. performed histological analysis. J.C., T.R.P., B.E.M., and J.M.P. wrote the manuscript. All authors reviewed and approved the manuscript.
