# Supplementary material for: Preoperative imaging of glioblastoma patients using hyperpolarized 13C pyruvate: Potential role in clinical decision making
Source: Neurooncol Adv. 2021 Jun 28;3(1):vdab092. doi: 10.1093/noajnl/vdab092 (PMC8331053; doi:10.1093/noajnl/vdab092)
Supplement: vdab092_suppl_Supplementary_Materials [file vdab092_suppl_supplementary_materials.docx]

**Supplementary Material**

**Supplementary Figures**

**Figure S1. Preoperative ^1^H MRI of patient #2 from the clinic.** CE T_1_w and perfusion images, which were omitted during the research hyperpolarized MR session due to the limited scan time, from the clinic. (A) CE T_1_w image of the tumor-bearing slice from patient 2. The red contour indicates the enhancing region. (B) Relative cerebral blood volume (rCBV) map acquired from dynamic susceptibility contrast (DSC) MRI. (C) T_2_w image. The yellow contour indicates the hyperintense region.

**Supplementary Tables**

|  | ROI | Relative lactate | | | | Relative bicarbonate | | | |
| --- | --- | --- | --- | --- | --- | --- | --- | --- | --- |
|  |  | Tumor | | NAB | | Tumor | | NAB | |
|  |  | Inj #1 | Inj #2 | Inj #1 | Inj #2 | Inj #1 | Inj #2 | Inj #1 | Inj #2 |
| Patient 1 | ROI1, 2 | 0.315 | 0.321 | 0.262 | 0.284 | 0.064 | 0.059 | 0.128 | 0.122 |
|  | ROI3, 4 | 0.298 | 0.333 | 0.248 | 0.237 | 0.113 | 0.135 | 0.118 | 0.126 |
| Patient 2 | ROI1, 2 | 0.154 | 0.166 | 0.143 | 0.149 | 0.070 | 0.080 | 0.094 | 0.089 |
| Patient 3 | ROI1, 2 | 0.286 | 0.284 | 0.238 | 0.216 | 0.111 | 0.102 | 0.111 | 0.107 |
|  | ROI3, 4 | 0.304 | 0.333 | 0.268 | 0.277 | 0.083 | 0.092 | 0.077 | 0.092 |

**Table S1. Comparison of hyperpolarized ^13^C metabolite levels between two injections of pyruvate.** ROI, region of interest; NAB, normal-appearing brain; Inj, injection.
